# Paradoxical dyshidrosis following bimekizumab treatment for severe recalcitrant psoriasis

**DOI:** 10.1016/j.jdcr.2025.10.062

**Published:** 2025-11-07

**Authors:** Anagha B. Thiagarajan, Luke Horton, Bonnie A. Lee

**Affiliations:** a Department of Dermatology, University of California, Irvine, Irvine, California; b Department of Dermatology and Dermatologic Surgery, Medical University of South Carolina, Charleston, South Carolina

**Keywords:** immune shift, paradoxical eczema, psoriasis

## Introduction

Psoriasis is a chronic, immune-mediate skin disorder characterized by erythematous, scaly plaques with micaceous scale.^1^ Psoriasis has several clinical variants driven, to some extent, by Th1 and Th17 immune pathway dysregulation. Acrodermatitis continua of Hallopeau (ACH) is a localized form of pustular psoriasis that often arises in individuals with an underlying history of plaque psoriasis.^2^ It predominantly presents with recurrent sterile pustules, scaling, and nail involvement that can progress to anonychia or osteolysis. Although ACH shares key inflammatory mechanisms with plaque psoriasis, it is typically more recalcitrant to treatment and prone to chronic relapse, highlighting the clinical and therapeutic continuum between these 2 entities.^2^ Recently, IL-17 inhibitors (IL-17i) have transformed the landscape of psoriasis treatment.^3^ However, potent IL-17 blockade can trigger immune class switching from Th1-driven psoriatic phenotypes to Th2-mediated atopic phenotypes.^4^ Sometimes referred to as “paradoxical eczema,” this phenotype switching has been documented with various IL-17i, such as ixekizumab, secukizumab, and brodalumab.^5^ We present one of the first described cases of a patient with recalcitrant psoriasis, complicated by ACH, who developed dyshidrotic eczema following IL-17i therapy with bimekizumab, consistent with a Th-1 to a Th-2 immune response deviation.

## Case report

**Fig 1:**
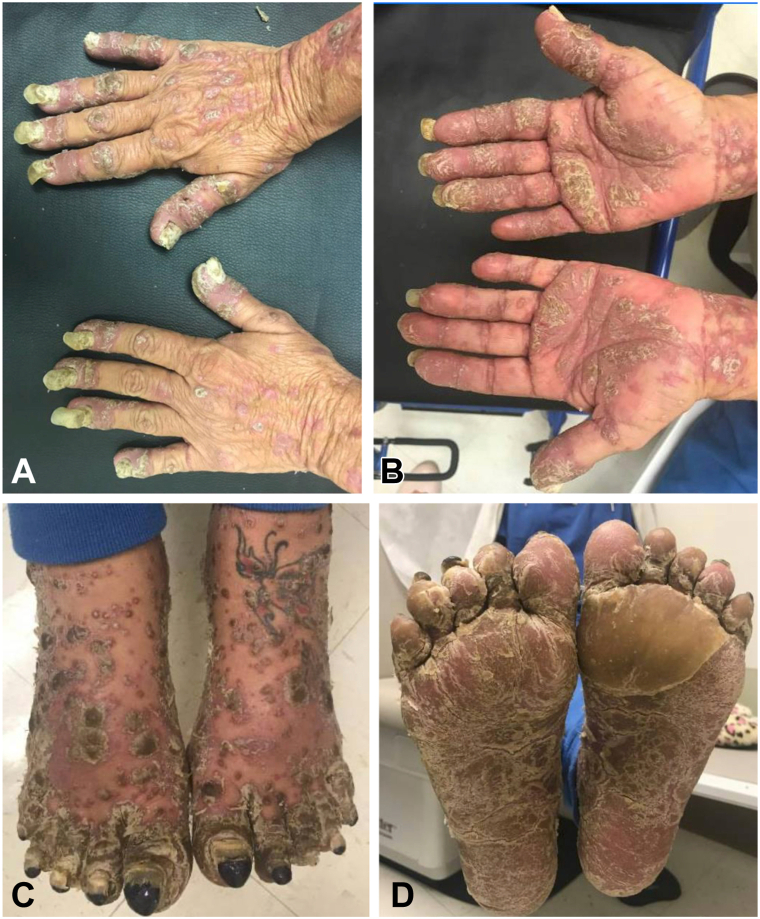
**A-D,** Recalcitrant psoriasis after failed treatments with several biologics prior to initiating bimekizumab. **A,** Dorsal hands. **B,** Palmar hands. **C,** Dorsal feet. **D,** Plantar feet.

**Fig 2:**
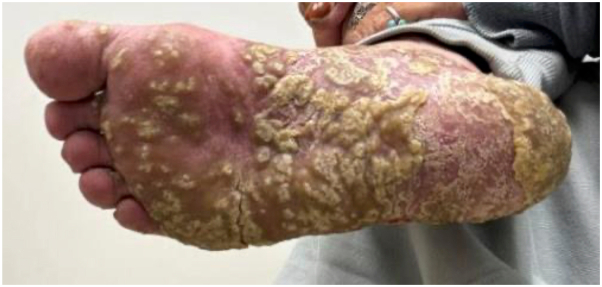
Flare of recalcitrant psoriasis on the plantar feet of prior to treatment with bimekizumab.

A 64-year-old female with a history of chronic hepatitis C presented with a 10-year history of recalcitrant psoriasis and psoriatic arthritis requiring multiple prior hospitalizations. Her family history was notable for a sister with Behcet disease. Her initial disease was characterized by thick psoriatic plaques involving over 85% of her body surface area, complicated by episodes of sterile pustules coalescing into lakes of pus on her hands and feet leading to the loss of her nails, consistent with ACH. In our clinic she had hyperkeratotic plaques on bilateral feet and hands as well as diffuse involvement of her body (Figs 1, *A*-*D* and 2). scoring 18.0 points on the Psoriasis Area and Severity Index (PASI).^6^ She had tried and failed multiple prior therapies including methotrexate, acitretin, tofacitinib, apremilast, and a range of biologic therapies including TNF alpha inhibitors (etanercept, adalimumab and infliximab), IL-23 inhibitors (ustekinumab, tildrakizumab, and guselkumab), IL-17 inhibitors (secukinumab and ixekizumab), and an IL-36 inhibitor, spesolimab. Treatment courses were administered for at least 3 months to a year to assess efficacy, with none resulting in paradoxical eczema.

**Fig 3:**
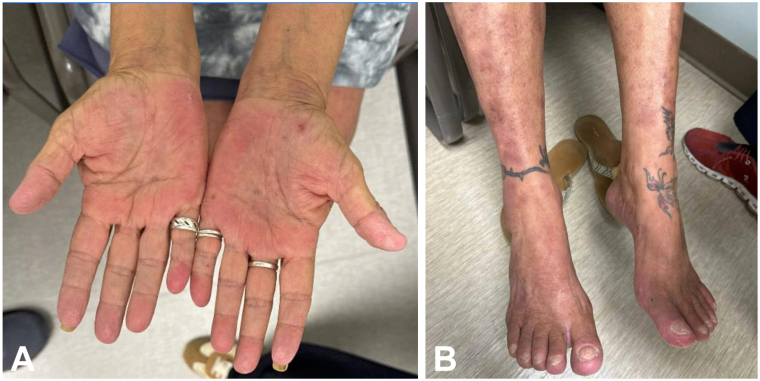
**A** and **B,** Resolution of recalcitrant psoriasis after 8 weeks of treatment with bimekizumab. **A,** Palmar hands. **B,** Dorsal feet.

**Fig 4:**
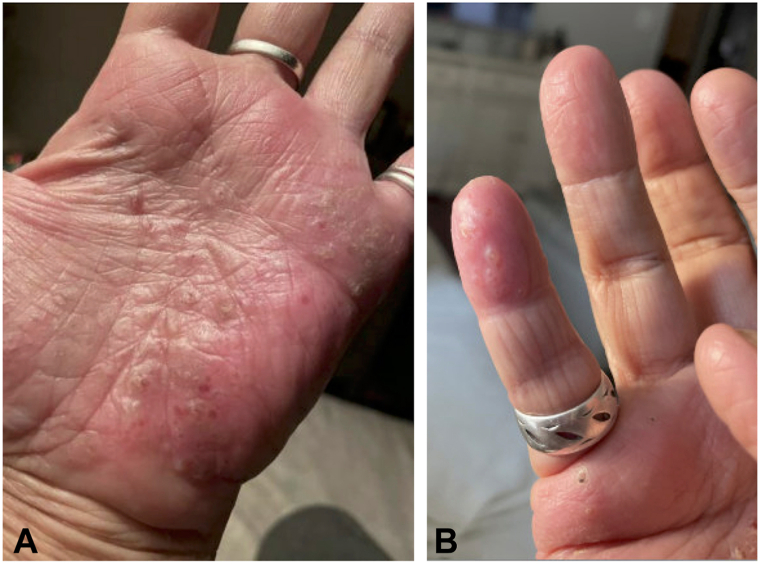
**A** and **B,** Vesicular flare of dyshidrotic eczema on the palmar hands against a smooth background after resolution of psoriatic lesions 5 months after treatment with bimekizumab. **A,** Palmar aspect of the hand. **B,** Palmar aspect of the fifth finger.

Given her recalcitrant disease, the patient began bimekizumab, with an initial 320 mg subcutaneous injection followed by 160 mg subcutaneous injections every 4 weeks, resulting in rapid improvement of her psoriatic lesions and arthritic pain (Fig 3, *A* and *B*). However, prior to her third dose after 8 weeks of bimekizumab therapy (2 doses), the patient presented with a new hand dermatitis characterized by more severe itching than experienced with her psoriasis. Physical exam at this time showed near complete resolution of her psoriasiform plaques; however, numerous new 2 to 4 mm pruritic clear fluid filled vesicles with overlying excoriations on the hands, wrists, and feet, amid a background of xerosis were appreciated (Fig 4, *A* and *B*). This clinical picture was compelling for dyshidrotic eczema and as such a biopsy was not pursued per patient preference. Topical betamethasone (0.05%) helped somewhat but the patient does not like using topical therapy on her hands. Acitretin (10 mg daily) was tried and failed due to medication intolerance. In addition, low dose methotrexate (10 mg weekly) was ineffective for her dyshidrotic eczema and was discontinued after a few months. At 16 months since initiating bimekizumab, the patient remains largely clear of psoriasis aside from a tiny periumbilical patch, and persistent nail dystrophy. She still occasionally experiences self-resolving pruritic blisters on her hands, but remains stable off methotrexate.

## Discussion

This is a rare case of phenotype switching from Th1 mediated psoriasis to Th2 mediated eczematous eruptions after bimekizumab treatment.

Bimekizumab, approved by the FDA in 2023, is a dual IL-17A/F inhibitor thought to offer a more potent IL-17 blockade compared to earlier agents targeting only 1 member of the IL-17 proinflammatory cytokine family.^7^ This increased potency may offer increased efficacy, though it may also induce immune phenotype switching as outlined in this case.^5^ While Th1 and Th2 pathways normally oppose each other, pharmacologically blocking 1 pathway (eg Th1) may allow the other pathway (eg Th2) to rebound as they are no longer counterbalanced by each other.^4^^,^^5^ This immune rebalancing is not a simple switch, but more of a redistribution of immune dominance, suggesting a mechanism for the development of paradoxical Th2-mediated disease while on IL17i therapy. Prior studies link increased age and female sex to an elevated risk for paradoxical eczema with IL-17i therapy.^4^^,^^5^ Immune class switching with other psoriasis biologics, such as TNF-alpha and IL-23 inhibitors, does occur, though it is documented less frequently than with IL-17i suggesting a less robust risk of immune deviation.^5^ Clinicians should have heightened awareness of paradoxical eczema when prescribing IL-17i therapies to patients, particularly in those with treatment refractory psoriasis.

This case also underscores a therapeutic dilemma, in that balancing effective treatments for refractory psoriasis against the emergence of new cutaneous reactions (eg eczematous eruptions) poses challenges for clinicians. In this patient, bimekizumab treatment was continued, despite the development of dyshidrosis, due to significant improvement of her recalcitrant psoriasis which had been plaguing her for decades. Still, managing the eczematous component of the patient’s symptoms proved to be challenging, as systematic sparing agents were either poorly tolerated (acitretin) or failed (methotrexate) at a lower dose given her chronic hepatitis C. Emerging literature suggests that various patient-specific factors such as serum cytokine levels, immune cell profiles, and skin transcriptome gene expression may help better understand the patient’s immune architecture, and contribute to more targeted and effective therapy.^8^

This case highlights a recalcitrant presentation of psoriasis that responded for the first time to IL-17A/F inhibition with bimekizumab. Notably, this response coincided with the patient’s first paradoxical Th2-mediated eruption despite prior exposure to nearly all available psoriasis biologics, raising the question of whether such immune shifts may be directly correlated with the potency of cytokine blockade. Future studies should aim to identify biomarkers that can aid in predicting such adverse responses, particularly in patients with multiple prior treatment failures.

## Conflicts of interest

None disclosed.
